# Computational RNomics of Drosophilids

**DOI:** 10.1186/1471-2164-8-406

**Published:** 2007-11-08

**Authors:** Dominic Rose, Jörg Hackermüller, Stefan Washietl, Kristin Reiche, Jana Hertel, Sven Findeiß, Peter F Stadler, Sonja J Prohaska

**Affiliations:** 1 Bioinformatics Group, Department of Computer Science, University of Leipzig, Härtelstraße 16-18, Leipzig, Germany, D-04107; 2 Fraunhofer Institute for Cell Therapy and Immunology, Deutscher Platz 5e, Leipzig, Germany, D-04103; 3 Interdisciplinary Center for Bioinformatics, University of Leipzig, Härtelstraße 16-18, Leipzig, Germany, D-04107; 4 Department of Theoretical Chemistry, University of Vienna, Währingerstraße 17,Wien, Austria, A-1090; 5 Biomedical Informatics, Arizona State University, Tempe, PO-Box 878809, USA, AZ 85287; 6 Santa Fe Institute,1399 Hyde Park Rd., Santa Fe, USA, NM 87501

## Abstract

**Background:**

Recent experimental and computational studies have provided overwhelming evidence for a plethora of diverse transcripts that are unrelated to protein-coding genes. One subclass consists of those RNAs that require distinctive secondary structure motifs to exert their biological function and hence exhibit distinctive patterns of sequence conservation characteristic for positive selection on RNA secondary structure.

The deep-sequencing of 12 drosophilid species coordinated by the NHGRI provides an ideal data set of comparative computational approaches to determine those genomic loci that code for evolutionarily conserved RNA motifs. This class of loci includes the majority of the known small ncRNAs as well as structured RNA motifs in mRNAs. We report here on a genome-wide survey using RNAz.

**Results:**

We obtain 16 000 high quality predictions among which we recover the majority of the known ncRNAs. Taking a pessimistically estimated false discovery rate of 40% into account, this implies that at least some ten thousand loci in the *Drosophila *genome show the hallmarks of stabilizing selection action of RNA structure, and hence are most likely functional at the RNA level. A subset of RNAz predictions overlapping with TRF1 and BRF binding sites [Isogai *et al*., *EMBO J*. 26: 79–89 (2007)], which are plausible candidates of Pol III transcripts, have been studied in more detail. Among these sequences we identify several "clusters" of ncRNA candidates with striking structural similarities.

**Conclusion:**

The statistical evaluation of the RNAz predictions in comparison with a similar analysis of vertebrate genomes [Washietl *et al., Nat. Biotech*. **23**: 1383–1390 (2005)] shows that qualitatively similar fractions of structured RNAs are found in introns, UTRs, and intergenic regions. The intergenic RNA structures, however, are concentrated much more closely around known protein-coding loci, suggesting that flies have significantly smaller complement of independent structured ncRNAs compared to mammals.

## Background

High-throughput transcriptome data obtained in particular using tiling arrays [1-6] and cDNA sequencing [7-9] in conjunction with detailed functional studies of individual genes have profoundly changed our picture of eukaryotic gene regulation by emphasizing multiple regulatory layers, many of which involve non-protein-coding RNAs (ncRNAs). In contrast to protein-coding genes, however, ncRNAs do not form a homogeneous group of transcripts but rather belong to a diverse array of classes with vastly different structures, functions, and evolutionary patterns [10-16].

Efficient computational methods [17,18] have recently been developed to determine the genomic inventory of a large subgroup of ncRNAs, namely those that exhibit evolutionarily conserved secondary structures. As stabilizing selection acts to preserve structure in the presence of sequence variation, these transcripts are very likely to have discernible biological function – as opposed to being a mere byproduct of transcriptional noise [19] or gene regulation by transcriptional interference [20]. The group of structured RNAs that we are considering here consequently includes the classical families of small ncRNAs (tRNAs, rRNAs, miRNAs, snRNAs, snoRNAs, RNAse P RNA, etc) as well as structured, usually regulatory, motifs associated with larger coding or non-coding transcripts, such as internal ribosomal entry sites, IRE, and SECIS signals see e.g. [21]. The RNAz approach [17] has proven to produce rather high quality predictions. In particular, as part of the detailed analysis of the ENCODE regions [22], the verification of many unannotated RNAz predictions by means of RT-PCR has been reported, and for a substantial fraction of RNAz predictions corroborating evidence from high-throughput experiments has been obtained.

Computational screens for structured RNAs have been reported so far for mammalian [22,23], urochordate [24], nematode [25], and yeast [26,27] genomes. However, no comprehensive analysis of structured ncRNAs in insect genomes has been published so far, even though there is statistical evidence for an enrichment of structured RNAs within highly conserved non-coding elements of drosophilids [28]. Drosophilids, which have been deeply sequenced by a consortium coordinated by the NHGRI [29-31], provide an ideal model system for this task, since their evolutionary divergence is comparable to those of mammals. As a consequence, large portions of their genomes are alignable, while at the same time there is substantial sequence variation. Both are necessary prerequisites for currently available ncRNA detection tools.

In addition to the statistical evidence for wide-spread structured RNAs in insects, two recent genome-wide experimental studies provide evidence of a large reservoir of novel ncRNAs in *Drosophila melanogaster*: Isogai *et al*. [32] mapped TRF1 and BRF binding sites in the *D. melanogaster *genome and showed that, unlike most other eukaryotes, TRF1/BRF binding appears responsible for the initiation of all classes of Polymerase-III (Pol III) transcription. As the known Pol III transcripts are small ncRNAs, their data suggests that drosophilids are likely to have a large set of previously unannotated small ncRNAs. A large-scale tiling array study of transcription in the early development of *D. melanogaster *[5] found that about 20% of the observed transcripts in *D. melanogaster *come from stand-alone intergenic or intronic sources and may constitute new types of RNAs, including a substantial fraction of ncRNAs.

## Results and Discussion

We report here on a computational screen for structured RNA motifs in Drosophilids based on 12-species Pecan alignments provided by the Consortium. The detected RNAz hits are either (parts of) independently transcribed non-coding RNAs with evolutionarily conserved secondary structures, or they are structured elements that are parts of coding transcripts such as SECIS or IRE elements.

### Sensitivity and Specificity

Overall, 42 482 RNAz hits corresponding to roughly 5 Mb in the *D. melanogaster *genome show evidence of evolutionarily conserved RNA secondary structure. About 20% of these overlap existing annotation. The 16 377 loci of the high confidence set covers approximately 2.1 Mb of DNA, see Tab. 1.

**Table 1:** Overall statistics of the RNAz screen. Initial filtering of Pecan alignments leaves roughly 50% as input for RNAz respectively to the ncRNA prediction. The distribution of RNAz hits does not show a chromosomal bias. We counted the number of predicted loci and their overall length at two probability thresholds (*p *> 0.5, *p *> 0.9) for normal and also randomized alignments. Obtained relative frequencies (given as percentages) can be interpreted as false discovery rates (FDR). As expected, the FDR decreases with a higher RNAz p-value.

	overall	chromosomes					
		2L	2R	3L	3R	4	X
alignments	4077	659	804	676	861	65	1012
aligned DNA [Mb]	117	22	21	23	28	1	22
screened by RNAz [Mb]	57.4	11	10	12	14	0.4	10
percentage	49	50	48	52	50	40	46
RNAz *p *> 0.5	42 482	7 824	6 646	8 765	10 351	196	8 700
[Kb]	5 079	927	783	1 060	1 229	25	1 055
RNAz *p *> 0.9	16 377	2 940	2 473	3 413	3 862	80	3 609
[Kb]	2 167	385	321	461	511	11	478
FDR *p *> 0.5 hits	56.5	54.5	57.2	57.5	55.9	68.4	57.3
sequence	52.8	50.7	53.6	53.9	52.4	64.0	53.0
FDR *p *> 0.9	45.3	43.6	45.1	47.8	46.2	43.7	43.8
sequence	40.2	38.2	40.2	42.5	41.1	36.4	38.7

In total, 336 hits correspond to known non-coding RNAs according to at least one source of annotation (FlyBase: 316; BLAST against miRBase: 79; BLAST against Noncode: 44; BLAST against Rfam: 222; tRNAscan: 159). Tab. 2 summarizes the recall of the screen on several "classical" ncRNAs families. Note that some classes of ncRNAs were deliberately removed already in the Pecan alignments, notably the 5S rRNA sequences. We recovered 96% of the known *D. melanogaster *miRNAs.

**Table 2:** Sensitivity of the RNAz screen on known ncRNAs.

Class	RNAz	input	annotated	sensitivity (%)	
tRNA	171	250	297	69	
5S rRNA	0	0	99	--	not in input
SRP RNA	0	0	2	--	not in input
RNAse P	1	1	1	100	
snRNA	18	22	22	81	U6 not detected
snoRNA	96	202	250	48	
miRNA	75	78	85	96	

A BLAST search of the drosophilid RNAz hits against the results of prior RNAz surveys of mammals [23], urochordates [24], and nematodes [25] yielded the following pattern of conservation: 167 tRNA hits and 11 snRNAs associated with the major spliceosome. Furthermore, we recover the U6atac snRNA (which was previously unannotated) and 5 microRNAs.

In order to estimate the false discovery rate (FDR), we repeated the screen with shuffled alignments as described in [33]. Alignments are shuffled such that two alignment columns are swapped only if both their gap pattern and their sequence conservation pattern is the same. This amounts to a very "gentle" shuffling that in particular preserves pairwise sequence divergence within any given window. This gentle shuffling procedure may fail to remove the secondary structure signal in some cases because too few pairs of alignment columns satisfy the stringent conditions for shuffling. This is at least one reason why we observe 3 239 (7.6%) hits in which true and shuffled screen intersect, including 53 of the 756 annotated *D. melanogaster *ncRNAs, almost exclusively tRNAs. The estimated FDR of roughly 50% for *p *> 0.5 and 40% for the high quality set in Tab. 1 should therefore be regarded as pessimistic estimates.

The results of a second, more "vigorous" shuffling approach lead to much more optimistic estimates: shuffling of the columns without considering their sequence conservation or gap pattern reduces the estimated FDR by factor of 35 to only a 1–2%. One may argue, of course, that shuffling columns independently will change the gap pattern of the alignment (even though it still conserves pairwise sequence identities). Hence, this procedure may well underestimate the FDR. The dramatic difference in the result highlights a general problem that so far has not been solved in a satisfactory way, namely how to systematically construct randomized *alignments *that preserve all correlation features of the genomic background except the one under consideration. One important feature which must be mentioned at this point is dinucleotide content. Due to the stacking energy contributions in the folding model, dinucleotide content can affect folding energies and thus FDR estimates considerably. Since there is still no way of randomizing alignments preserving dinucleotide content, we cannot control for this effect. However, we found that, in contrast to mammalian genomes [22], there is no strong dinucleotide bias in the genomic background of *D. melanogaster *that effects folding energies. Therefore, our estimates from mononucleotide shuffled alignments will not differ dramatically from estimates one would obtain from controls with the same dinucleotide content.

**Figure 1:**
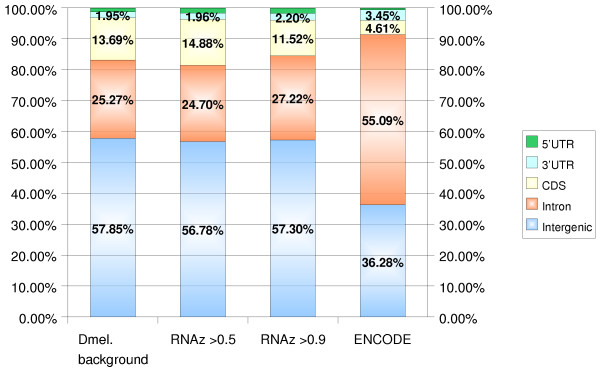
**Genomic distribution of D. melanogaster RNAz hits**. We compare genomic locations of the RNAz hits in *D. melanogaster *for two different classification thresholds with the corresponding distribution of the input alignments (relative to the current FlyBase gene track from the UCSC Table Browser, April 2004). In addition, the corresponding distribution for the human ENCODE regions [22] is shown. The numbers differ slightly from ref. [22] since here we have normalized them to 100%. Percentages for the 5'-UTRs are not given due to the very small bar areas; the values are (from left to right): 1.24%, 1.69%, 1.70% and 0.6%. In general, the distribution of structured RNAs closely follows that of conserved sequence, i.e., there is no strong enrichment of RNAz hits in a particular annotation class. The most striking difference between human and fly is the much larger fraction of intronic RNAz hits in the ENCODE data.

In contrast to most previous RNAz screens, we have not removed coding sequences from the input alignments. Notably, 8 021 hits for *p *> 0.5 and 2208 hits for *p *> 0.9 overlap with annotated coding regions, accounting for 19% and 13% of the RNAz hits, respectively. These fractions are much smaller than the expected FDRs; we therefore expect that most of these signals are indeed false positives. Interestingly, if we base our analysis on the number of nucleotides that are predicted to lie in regions with conserved structures instead of counting the RNAz hits the estimates are reduced to 15%, and 11.5%, respectively (cp. Fig. 1). Conversely, only 12% (8 326) at *p *> 0.5 and less than 4% (2 522) at *p *> 0.9 of the annotated coding regions are detected by RNAz. Note that 1 398 RNAz hits overlap more than one annotated coding region. The small percentage of RNAz hits in annotated CDS indicates that even a possibly large number of unannotated coding sequences will not have a significant impact on the interpretation of the RNAz results in the sense that only a small fraction of the RNAz hits may be previously unannotated CDS. To further corroborate this point we have computed the overlap of the RNAz predictions with various gene prediction tracks available in the UCSC Table Browser, yielding no significant increase in the number of RNAz hits located in putative CDS: In total only 11 172 (*p *> 0.5) and 3 144 (*p *> 0.9) RNAz hits lie in regions with any evidence for coding capacity.

### Genomic Distribution

The genomic distribution of structured RNA candidates in *D. melanogaster *is comparable to the observations in previous RNAz-based screens, see Fig. 1. As in the ENCODE data [22], the distribution of RNAz hits largely follows the patterns of sequence conservation. In the fly data, only 5'UTRs show a substantial enrichment relative to the input data. In contrast, the largest enrichment in the human ENCODE data was observed from 3'UTRs [22]. The most striking difference between fly and human data is that the relative fraction of both intronic RNAz hits and intronic sequence conservation is twice as large in human.

In a recent article Manak and colleagues [5] describe widespread transcriptional activity in the *D. melanogaster *genome during 12 timepoints of early embryonic development detected by genomic tiling arrays. When comparing the RNAz hits to this data, we identify 4 236 (*p *> 0.5) and 1 713 (*p *> 0.9) hits that overlap a Transfrag in any of the 12 timepoints. A comparison of the fractions of RNAz hits from normal and control screen which overlap Transfrags in one, several or all timepoints yields, however, no significant enrichments (see Additional file 1 for details).

**Figure 2:**
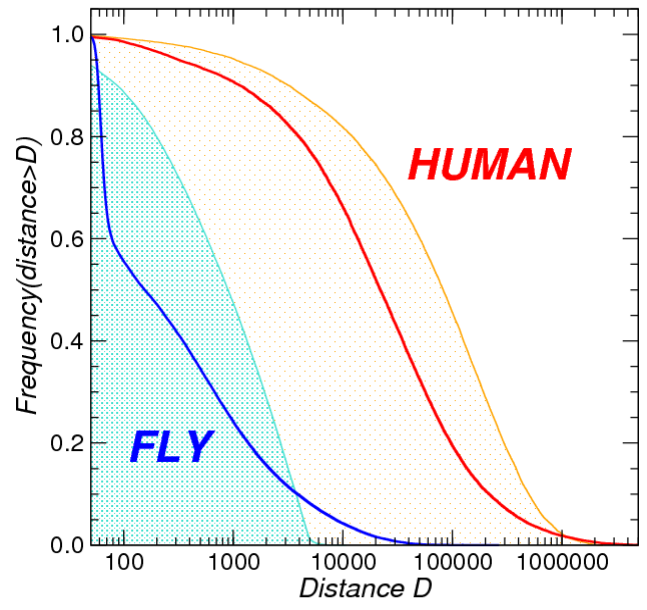
**Distributions of IGR length and distances of RNAz hits to their nearest annotated CDS element in fly and human**. The two curves with shaded backgrounds show the distribution of IGRs that exceed a given length *D *for *Homo sapiens *and *Drosophila melanogaster*, respectively. The shape of these curves is very similar. Note that, although distances *D *< 50 nt are omitted in the plot, all cumulative distributions of course reach 1 at *D *= 1. The main difference is that the IGRs in fly are on average two orders of magnitude shorter. Thick lines indicate the distribution of distances of RNAz hits that have a distance of more than *D *from the nearest coding sequence. In humans, this distribution is similar to the IGR distribution, shifted to the left by a factor of 3 to 4. In contrast, we observe a completely different shape in flies: A fraction of about 40% of the RNAz hits is located adjacent to the annotated genes. On the other hand, a small fraction of the RNAz hits is located further away from coding genes than expected. RNAz hits refer to the comprehensive set *p *> 0.5.

The distance distribution of intergenic RNAz hits reveals a striking difference between the situation in the human and the fly genome, Fig. 2. Since the *D. melanogaster *genome is much more compact than the human one, we need to compare the distribution of the distances between RNAz hits and the nearest coding sequence relative to the length distribution of the intergenic regions (IGR). In Fig. 2 we plot the relative frequency of IGR with a length exceeding a given distance *D*, and the relative frequency of RNAz hits with a distance larger than *D *from the nearest coding region. If intergenic RNAz hits are uniformly distributed within the IGR, the distribution of RNAz-CDS distances looks like the distribution of IGR distances, just shifted to the left by a factor of 4. Indeed, this is observed in the human data, albeit the shift is a factor between 3 and 4, indicating that the placement of intergenic RNAz hits in human is nearly uniform, with a small tendency of avoiding the proximity of coding genes.

In contrast, about 40% of the *D. melanogaster *RNAz hits in intergenic regions are located adjacent to coding sequences. This may indicate that current annotation of the fly genome lists boundaries of protein coding genes that systematically truncate the UTRs. If this is the case, however, then we would have to interpret more than 15% of the total RNAz hits as located in UTRs. Our data could be explained if a situation similar to the *minifly *gene is prevalent in the fly: For this gene a recent study [34] described several alternative poly-A sites and multiple small ncRNAs that are processed from the alternative 3'UTRs. At least one of these ncRNAs is structured: the snoRNA H1 was also detected in our screen. In any case, the structured RNAs by RNAz are on average much more closely linked to protein coding genes in flies than in human.

On the other hand, a small fraction (≈ 10%) of the intergenic RNAz hits, i.e., the tail in Fig. 2, is located much further away from CDS than expected for random placement. This suggests the existence of a distinct class of RNAz hits with a propensity for large IGRs. Most likely, these signals correspond to independently transcribed ncRNAs.

About 20% of the unannotated transcripts observed in *D. melanogaster *early development arise from stand-alone intergenic or intronic sources (relative to FlyBase annotation) [5]. Only a relatively small fraction of the novel independent transcripts (5.1% of the total transcriptional output) had intergenic origin. In comparison, more than 13% [21.9% of 60%] of the transcriptional output recorded by comparable methods from the ENCODE regions has a distal intergenic source (in relation to annotated exons) [22]. This difference is in agreement with closer association of most RNAz hits with protein coding genes in the fly.

### Further Annotation of RNAz Predictions

In a recent study, Isogai *et al*. [32] identified TRF1 and BRF binding sites using high-resolution genome tiling microarrays and provided evidence that in *Drosophila *the alternative TRF1/BRF complex appears responsible for the initiation of all known classes of Pol III transcription. At the *p *> 0.9 significance level RNAz hits are about three-fold enriched in these regions. We have therefore analyzed the distribution of RNAz hits within the experimentally determined TRF1 and BRF binding regions. As reported in [32], most of the sites correspond to tRNAs, 7SL RNAs, and a subset of snoRNAs. In addition to these known ncRNAs, the loci contain 197 unannotated RNAz hits, which are prime candidates for novel Pol III transcripts.

In order to identify putative microRNAs, we screened all RNAz hits with RNAmicro [35]. This results in 607 candidates, of which 541 are unannotated so far. 176 of these signals are located in annotated CDS and are therefore most likely false positives, leaving 365 plausible microRNA candidates. The recent discovery of hundreds of new human microRNAs that are not conserved beyond primates strongly suggests that "evolution of miRNAs is an ongoing process and that along with ancient, highly conserved miRNAs, there are a number of emerging miRNAs" [36]. In the light of these data, a large number of drosophilid-specific microRNAs does not come unexpected.

Using SnoReport [58],  RNAz hits are classified as putative box H/ACA snoRNAs, of which 4 intersect with previously annotated snoRNAs. Taking into account that for only 22 of the 250 annotated snoRNAs the annotation distinguishes between box H/ACA (3), box C/D (18), and scaRNAs (1), the small overlap with the existing annotation is not surprising. Again, recent experimental surveys in other species, including nematodes [37,38] and mammals [39] have discovered a substantial number of previously unannotated snoRNAs in these species, suggesting that the current annotation of snoRNAs in *D. melanogaster *is also far from complete.

Finally, 1 700 RNAz hits have direct evidence for expression through ESTs that are not related to protein coding genes, i.e., through ESTs that do not intersect with the FlyBase, RefSeq, N-SCAN, Genscan, Human Proteins gene prediction and mRNA tracks of the UCSC Table Browser.

### Structure-Based Clustering

Since the 197 RNAz hits that overlap TRF1 or BRF binding regions [32] are good candidates without annotation for *bona fide *ncRNAs, we applied structure-based clustering to this small subset of our predictions to identify common secondary structures and, hence, putative novel functional RNAs. The complete clustering tree as well as a table of the most prominent clusters is given in the Additional file 1. Since all clusters have a mean pairwise identity less than 45%, structurally related candidates are typically highly diverged at sequence level.

**Figure 3:**
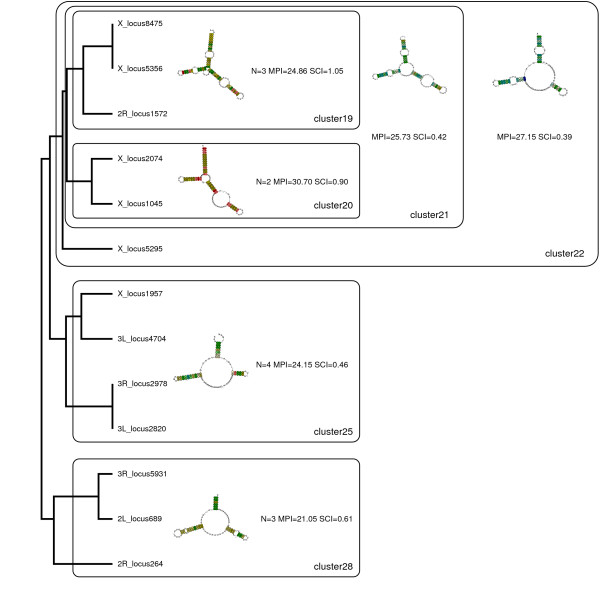
**Cluster of complex structures**. Structure-based clustering of RNAz hits with evidence for transcription by Pol III identifies a group of *Y *-shaped, potentially related putative ncRNAs. Abbreviations: N...number of sequences in cluster. MPI...mean pairwise identity of multiple alignment. SCI...structure conservation index.

In Fig. 3 an example cluster of complex structures is given. Clusters 22, 25 and 28 have a structure with two stem loops in common. All consensus structures show compensatory mutations.

**Figure 4:**
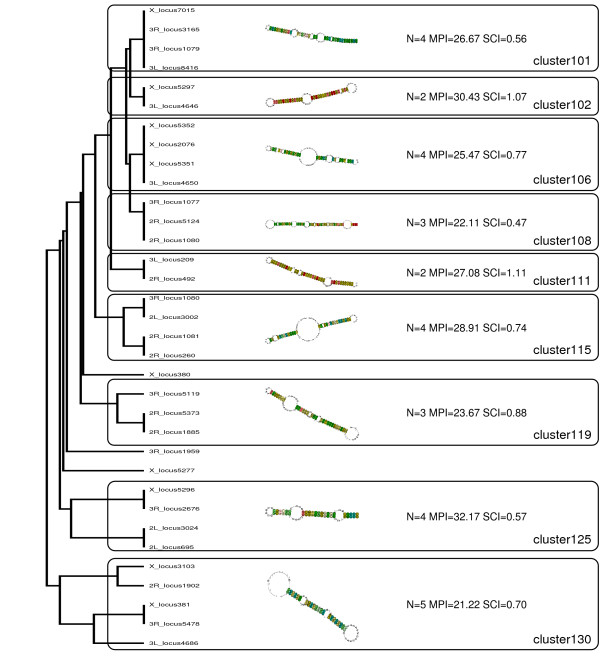
**Cluster of simple hairpin structures**. A fraction of the RNAz hits with evidence for transcription by Pol III exhibits hairpin structure. However, they lack any other annotation. This is in line with the finding that miRNAs are not transcribed by Pol III. Abbreviations: N...number of sequences in cluster. MPI...mean pairwise identity of multiple alignment. SCI...structure conservation index.

Fig. 4 depicts a large cluster of simple hairpin structures. They show a relatively high structural conservation (high structure conservation index, SCI) whereas the sequence similarity (expressed as main pairwise identity, MPI) is small.

### Phylogenetic Distribution

**Figure 5:**
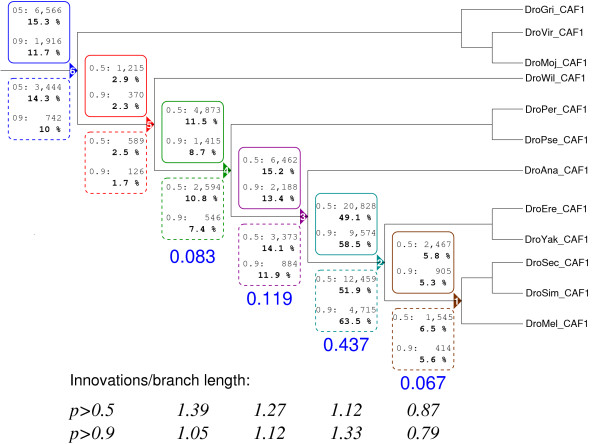
**Phylogenetic distribution of ncRNA candidates**. The tree only represents the topology and is not drawn to scale. Branch lengths are indicated below by large numbers in sans serif font, measured in terms of substitutions per site for 4-fold degenerated sites. For each branch we mark the number of RNAz hits for *p *> 0.5 and *p *> 0.9, respectively above the branch leading to the last common ancestor (LCA) of the sequences in the corresponding input alignment (full boxes). Below the branches we indicate the corresponding numbers for the "gentle" control screen. Below the tree the ratio of the fraction of newly appearing RNAz hits and the branch length is given, indicating little variation in the "innovation rate". Since the original tree is unrooted without an outgroup, no data are available for the branch separating *Sophophora *from the rest. The number of RNAz hits listed in the tree is smaller than the total number of RNAz hits because we only considered sequences present in all single windows of an RNAz hit here.

In order to study the phylogenetic distribution of the RNAz prediction we determine the last common ancestor for each RNAz hit that contains the corresponding sequence in the input alignment. Fig. 5 summarizes these results for both the true data and the "gentle" control screen.

More than 50% of the RNAz hits are found only within the *melanogaster *subgroup. To interpret this result we compute the ratio of newly appearing RNAz hits and the branch length for each branch in the tree leading to *D. melanogaster*. We observe little variation in the data, with the exception of a reduced rate of innovation along the most recent branch. This reduction is, however, most likely a methodological artefact, since the pairwise mutation distances between *D. melanogaster*, *D. simulans*, and *D. sechellia *are only about 0.1 and RNAz is known to be less sensitive for highly similar sequences.

Approximately 12% of the RNAz hits are conserved throughout all drosophilids. In comparison, a screen of vertebrate genomes [23] found about 3% of mammalian RNAz candidates (1 000 out of 36 000) to be conserved throughout vertebrates.

A comparison of true and shuffled screens furthermore indicates a small but significant decrease of the FDR with phylogenetic age of the RNAz hit.

## Conclusion

The present computational survey of drosophilid genomes yields about 16 000 high quality predictions. Taking into account the (very pessimistically estimated) false discovery rate of about 40%, this implies that at least some ten thousand loci in the *Drosophila *genome show the hallmarks of stabilizing selection acting on RNA structure, and hence are most likely functional at the RNA level. The elucidation of these functions, however, remains elusive in many cases. Here, we have studied a small subset in more detail. Almost 200 RNAz hits overlap with loci that are likely to be transcribed by Pol III, strongly suggesting that these are *bona fide *ncRNAs. Using structural clustering, we discovered several groups of structural similar ncRNA candidates in these regions.

This number of putative ncRNAs and a regulatory RNA element is not unexpected given that about 36 000 high quality RNAz hits have been found by a similar procedure in a screen of mammalian genomes [23], which was based on a comparable size of the input set comprising about 103 Mb of the human genome and a similar number of putative ncRNAs was reported using the SCFG-based evofold approach [18].

A comparison with the results from a similar RNAz screen of the human genome [17] and with an analysis of ENCODE regions [22], shows many similarities and several striking differences. We observe a smaller fraction of intronic and larger fraction of protein coding hits (cp. Fig. 1) in flies. A comparison of the distances between RNAz hits and their nearest annotated protein coding sequence shows that structured RNAs are concentrated much more strongly around known genes in flies than in human, even when accounting for the much more compact *D. melanogaster *genome. This observation agrees with recent tiling array data [5] which showed that a much smaller fraction of intergenic transcription is truly independent from surrounding protein coding genes in flies compared to human [6].

The inventory of structurally conserved RNAs is only a very small subset of the total non-coding transcriptional output, which covers most of the non-repetitive genome [5]. The current computational approach relies on substantial sequence conservation. Indeed many of the known ncRNAs that were missed in our survey were not in the input set. In fact, RNAz explicitly requires two independent signals for stabilizing selection: (1) sequence conservation so that a good alignment can be computed as input, and (2) stabilizing selection on RNA secondary structure in the presence of sequence variation. RNAz hits are therefore subject to specific selection pressures that make it highly likely that RNAz predictions have distinctive biological function. In contrast, it has been shown recently, that in some cases, such as the bithoraxoid ncRNAs of the *Drosophila *bithorax complex, ncRNA transcription itself, acting in cis, represses a target gene (in this case Ubx) [20]. In such a scenario, however, we do not expect to observe high levels of sequence conservation of the non-coding transcripts or the tell-tale substitution patterns of conserved secondary structures.

## Methods

### Data Sources

For our analysis we used the Pecan [40] alignment of the 12 drosophilid genomes [30,31] of the Comparable Analysis Freeze 1 (CAF1, Feb. 2006). The alignments were downloaded from [31,41]. We favored the Pecan alignments over two other sets of drosophilid alignments that are available at [31,42]. Visual inspection strongly suggested that Mavid alignments [43,44] are more biased towards protein coding regions. We did not use the Multiz alignments, because they contain three additional genomes (insects), and removing those sequences would effectively require a complete realignment in order to obtain a fair comparison between screens performed on different input alignments. The Pecan alignments comprise the *D. melanogaster *chromosomes 2L (22.4 Mb), 2R (20.8 Mb), 3L (23.8 Mb), 3R (27.9 Mb), 4 (1.3 Mb), and X (22.2 Mb).

### Preprocessing of Input Alignments

The current implementation of RNAz is restricted to input alignments containing at most 6 sequences and a maximum length of 400 nt due to the training of the underlying SVM [17]. In addition, certain restrictions apply for the fraction of gaps and on the overall base composition as a consequence of the data sets that were used to train the SVM model. The original genomic alignments thus need to be re-processed. The protocol used in this contribution closely follows that of previous RNAz-based studies:

Alignments longer than 120 nt are cut into 120 nt slices in 40 nt steps, so that subsequent slices overlap in 80 nt. This default length is motivated by the fact that many structured RNAs are less than 100 nt long. Such short signals would "drown" in the noise of longer alignments that are then mostly unstructured. On the other hand, alignments that are too short do not yield reliable signals for secondary structure conservation. In a series of filtering steps, sequences were removed from the individual alignments or alignment slices if they are (a) shorter than 50 nt, or (b) contain more than 25% gap characters, or (c) have a base composition outside the definition range of RNAz (e.g. GC content > 0.75 or < 0.25).

Alignments were discarded completely if fewer than 3 sequences were left after the filtering steps, or they did not contain a *D. melanogaster *sequence, since this species serves as a reference and as the basis for subsequent annotation. All preprocessing steps were performed using the script rnazWindows.pl of the current release of the RNAz package [45].

For alignment slices with more than 6 sequences, rnazWindows.pl selects a representative subset consisting of the *D. melanogaster *sequence and five additional sequences in such a way that 6 sequences are as evenly distributed in the dataset as possible and approach an average pairwise sequence identity of 80%, the optimal working range of the RNAz program. In practice that means that only a single representative from nearly identical sequences is chosen, and highly divergent sequences are excluded provided there is sufficient sequence variation in the remaining alignment. For the technical details of the procedure we refer to the documentation of the RNAz package .[45]

Tab. 1 summarizes the initial filtering steps. Roughly 50% of the nucleotides in the Pecan alignments are still contained in the RNAz input data.

### RNAz Classification and Annotation

RNAz was applied to the filtered input alignment slices in both reading directions. Overlapping slices with a positive ncRNA classification probability of *p *> 0.5 were combined using rnazCluster.pl to a single annotation element, which we will refer to as *"RNAz hit"*. From these data, we extract a subset of high confidence RNAz hits that contain at least one slice with a prediction confidence of *p *> 0.9.

In order to estimate the false discovery rate (FDR) of the screen we repeated the entire procedure with shuffled input alignments as described in [33]. The alignments were (1) shuffled using the rnazRandomizeAln.pl script (part of the RNAz package). It wraps a conservative shuffling procedure that maintains local characteristics of an alignment, e.g. columns with the same gap and conservation pattern. All remaining RNAz hits of this control screen are then shuffled once again (2) using a more stringent shuffling method that explicitly shuffles all columns of a given alignment randomly (cp. Tab. 3).

**Table 3:** Numbers of positive scored RNAz windows of the control screen.

shuffling method	chromosomes						
	all	2L	2R	3L	3R	4	X
conservative	29 938	5 220	631	6 402	7 254	160	6 271
complete	662	123	99	132	155	1	152

The RNAz hits were annotated using the *D. melanogaster *sequence as reference. We performed the following annotation steps:

• **Overlap with known *D. melanogaster *annotation**

We used the coordinates of a set of *D. melanogaster *non-coding RNAs, publicly available as gff files at [46] and [31,47] to identify already known *D. melanogaster *ncRNAs among our predictions. Furthermore, we computed the overlap of the RNAz hits with the CDS annotations from [31,48] (file = dmel-all-r4.3.filtered.gff) and [49] (file = all_caf1_DGIL_TEX.gff).

• **Overlap with public non-coding RNA databases**

We furthermore performed BLAST [50] searches using rnazBlast.pl against the Rfam (version 7.0) [51], Noncode (version 1.0) [52], ncRNAdb [53], FlyBase (version 2006 00.2 Beta) [54,55], and miRBase (version 9.0) [56].

• **Tools for annotation of specific RNA families**

We furthermore used tRNAscan [57] to annotate tRNAs and RNAmicro [35] to classify putative microRNAs. RNAmicro is an SVM based classification method that evaluates both thermodynamic stability and evolutionary conservation patterns.

We used SnoReport to recognize putative snoRNAs (for technical details see [58]). Similar to RNAmicro, SnoReport is composed of a pre-filter, a secondary structure prediction step, and a subsequent SVM-based classificator. In brief, the prefilter searches for consecutive H (pattern: ANANNA) and ACA boxes [59]. In the second step, the constraint folding option of RNAfold [60] is used to compute the secondary structure subject to the constraint that both boxes remain unpaired. If this results in an snoRNA-like secondary structure, several sequence and structure features are computed and passed to an SVM for classification. The model was trained on the set of snoRNAs that can be downloaded from the snoRNABase [61]. C/D box and scaRNAs represented the negative and H/ACA box snoRNA sequences the positive samples. Estimated positive and negative prediction values for the model used here are 80% and 99.9%, respectively.

Structure-based clustering was performed as described in [62]: The modified Sankoff algorithm implemented in the LocARNA program is used to compute local structural alignments and their consensus structure. The clustering tree is obtained by agglomerative clustering using LocARNA alignment scores as distance measures. To avoid that large scores influence the distance transformation we define distances by *d*(*i*, *j*) = *max*(0; *q *- *score*(*i*, *j*)), where q is here the 99% quantil of all pairwise scores. Since the procedure is computationally very demanding we have restricted this type of analysis here to a small subset of RNAz hits that are likely Pol III transcripts.

The phylogenetic relationships within drosophilids are taken from the AAA (Alignment/Analysis/Annotation of 12 related *Drosophila species*) web site [63]. Branch lengths are genomic mutation distances computed from 4-fold degenerate sites in all coding regions corrected for base composition as in [31][64]. In order to determine the branch in the phylogenetic tree at which an RNAz hit first appears, we determine the last common ancestor (LCA) of the sequences in the corresponding input alignment and assign the RNAz hit to the branch in the tree leading to this internal node. Due to the fact that RNAz hits are a combination of single windows and each window represents a specific selection of sequences out of an n-way alignment, we only considered those sequences for the LCA analysis which are simultaneously present at all windows.

## Authors' contributions

DR performed the RNAz screens and coordinated the analysis; SJP initiated this study. All authors closely collaborated in annotation, statistical analysis, and interpretation of the data, contributed to writing and approved the final manuscript.

## Additional Files

Machine readable annotation files and annotation tables for all RNAz predictions can be found at [65]. Supplemental figures and tables are appended as separate PDF file (Additional file 1).

## Supplementary Material

Additional file 1 Supplemental figures and tables. Figure 1: The *D. melanogaster *antennapedia complex. Figure 2: The *D. melanogaster *bithorax complex. Figure 3: Exemplary consensus secondary structures of two RNAz predictions. Figure 4: Comparison of obtained p-values. Figure 5: Complete WPGMA cluster tree of RNA candidates overlapping TRF and BRF binding regions. Table 1: Comparison of RNAz predicted ncRNAs using normal and randomized alignments. Table 2: Summary of RNAz predicted ncRNAs. Table 3: Number of predicted ncRNAs which overlap with Transfrags from [5]. Table 4: Number of predicted ncRNAs which overlap with Transfrags from [5] in one, several or all timepoints. Table 5: Intersection (> 80%) of RNAz predictions and UCSC Table Browser tracks. Table 6: Most prominent structural clusters of novel RNA candidates that overlap TRF or BRF binding regions. Click here for file
