# Supplementary material for: Computational RNomics of Drosophilids
Source: BMC Genomics. 2007 Nov 8;8:406. doi: 10.1186/1471-2164-8-406 (PMC2216035; doi:10.1186/1471-2164-8-406)
Supplement: Additional file 1 — Supplemental figures and tables. Figure 1: The D. melanogaster antennapedia complex. Figure 2: The D. melanogaster bithorax complex. Figure 3: Exemplary consensus secondary structures of two RNAz predictions. Figure 4: Comparison of obtained p-values. Figure 5: Complete WPGMA cluster tree of RNA candidates overlapping TRF and BRF binding regions. Table 1: Comparison of RNAz predicted ncRNAs using normal and randomized alignments. Table 2: Summary of RNAz predicted ncRNAs. Table 3: Number of predicted ncRNAs which overlap with Transfrags from [5]. Table 4: Number of predicted ncRNAs which overlap with Transfrags from [5] in one, several or all timepoints. Table 5: Intersection (> 80%) of RNAz predictions and UCSC Table Browser tracks. Table 6: Most prominent structural clusters of novel RNA candidates that overlap TRF or BRF binding regions. [file 1471-2164-8-406-S1.pdf]

Computational RNomics of Drosophilids
# - SUPPLEMENT -

Dominic Rose[a], Jörg Hackermüller[a,b], Stefan Washietl[d], Sven Findeiß[a], Kristin Reiche[a], Jana Hertel[d,a], Peter F. Stadler[a,c,b,d,f], and Sonja J. Prohaska[e]

{dominic, joerg, kristin, jana, studla}@bioinf.uni-leipzig.de
{wash, jana, studla}@tbi.univie.ac.at
sonja.prohaska@asu.edu

(a) *Bioinformatics Group, Department of Computer Science, University of Leipzig, Härtelstraße 16-18, D-04107 Leipzig, Germany*

(b) *Fraunhofer Institute for Celltherapy und Immunology, Deutscher Platz 5e, D-04103 Leipzig, Germany*

(c) *Interdisciplinary Center for Bioinformatics, University of Leipzig, Härtelstraße 16-18, D-04107 Leipzig, Germany*

(d) *Department of Theoretical Chemistry University of Vienna, Währingerstraße 17, A-1090 Wien, Austria*

(e) *Biomedical Informatics, Arizona State University, Tempe, PO-Box 878809, AZ 85287, USA Arizona 85287*

(f) *Santa Fe Institute, 1399 Hyde Park Rd., Santa Fe, NM 87501, USA*

May 11, 2007

This document contains supplementary supplementary tables and figures.

Visit
`http://www.bioinf.uni-leipzig.de/Publications/SUPPLEMENTS/07-001/`
for additional material.

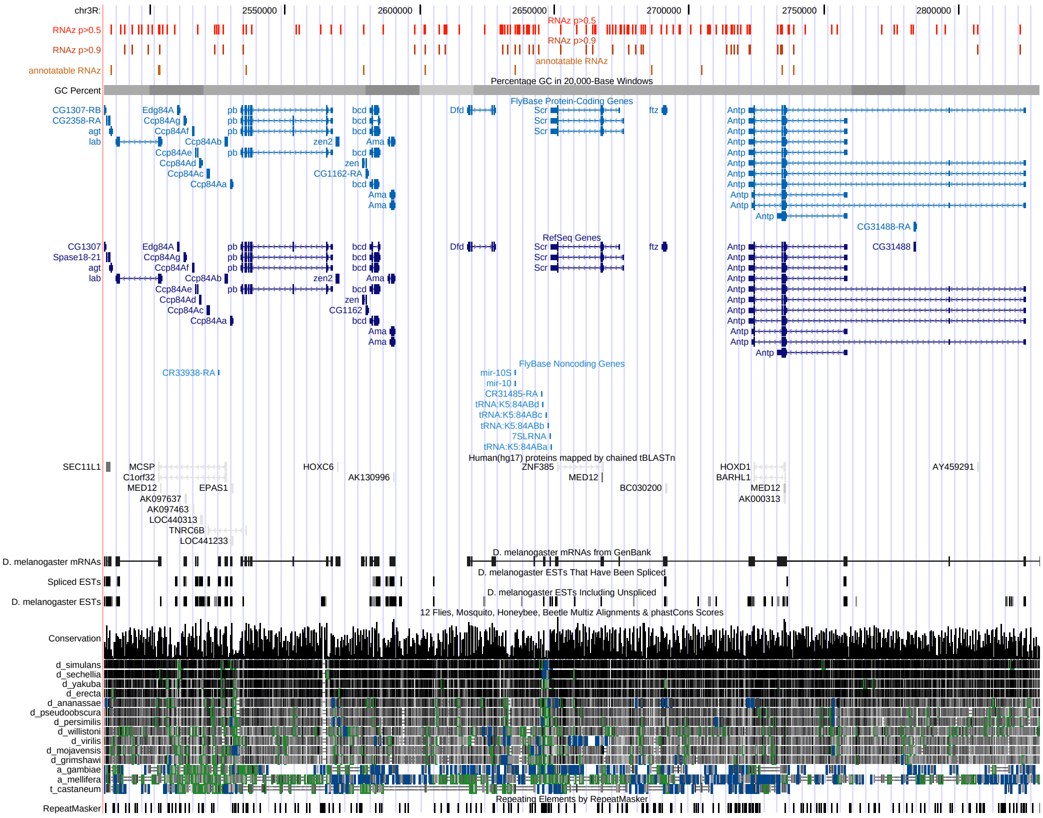

Figure 1: *D. melanogaster* antennapedia complex.
The figure representing the *D. melanogaster* genomic region 2 483 000 to 2 830 000 comprises the Antennapedia complex of chromosome 3R. This prominent drosophilid hox cluster includes the labial (lab), proboscipedia (pb), zerknullt (zen), bicoid (bcd), Deformed (Dfd), Sex combs reduced (Scr), fushi tarazu (ftz) and Antennapedia (Antp) genes. We incorporated all `RNAz` predicted ncRNA loci exceeding the two p-value thresholds 0.5 and 0.9 and, additionally, the set of annotatable predictions (red). A comparison with `FlyBase` ncRNAs, for example, reveals, that we hit the mir-10 miRNA. Located at 2 648 220-2 648 518 the picture shows the 299 nt long 7SL RNA (`FlyBase` annotation track). We could not recover this ncRNA, because the `Pecan` alignment of this region only consists of two sequences (*D. melanogaster* and *D. simulans*). Hence, the alignment was not screened.

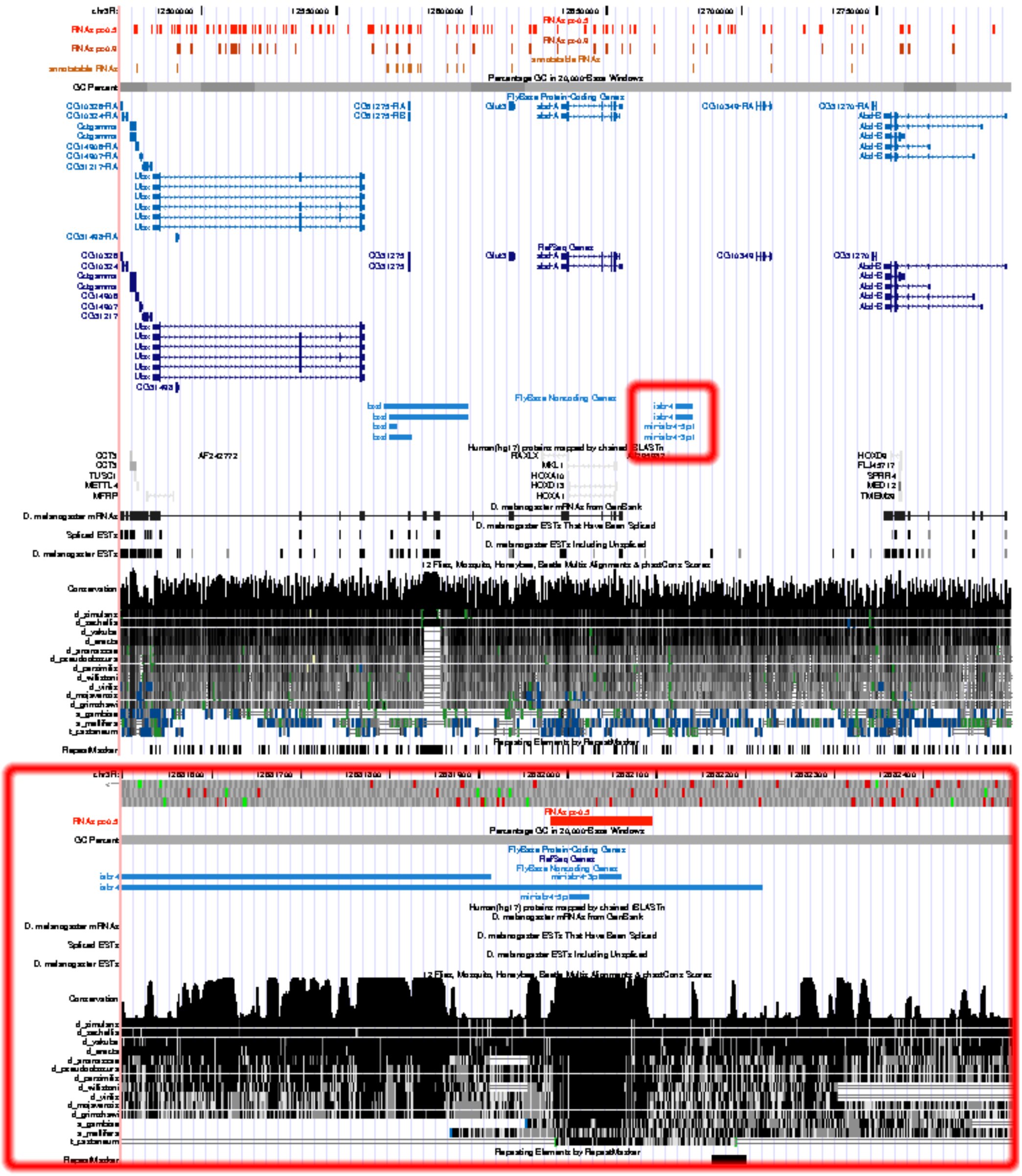

Figure 2: *D. melanogaster* bithorax complex.
The figure representing the *D. melanogaster* genomic region 12 470 000 to 12 800 000 comprises the bithorax complex of chromosome 3R. This prominent drosophilid hox cluster includes the Ultrabithorax (Ubx), abdominal-A (abd-A) and Abdominal-B (Abd-B) genes. We incorporated all `RNAz` predicted ncRNA loci exceeding the two p-value thresholds 0.5 and 0.9 and, additionally, the set of annotatable predictions. A comparison with `FlyBase` ncRNAs, for example, reveals, that we hit the miRNAs mir-iab-4-3p and mir-iab-4-5p. A detailed view (red rectangle) of this miRNA cluster is available at the bottom of the figure. It represents the *D. melanogaster* genomic region 12 470 000 to 12 800 000 of chromosome 3R illustrating the `RNAz` locus 4842, the recovered, already annotated mir-iab-4.

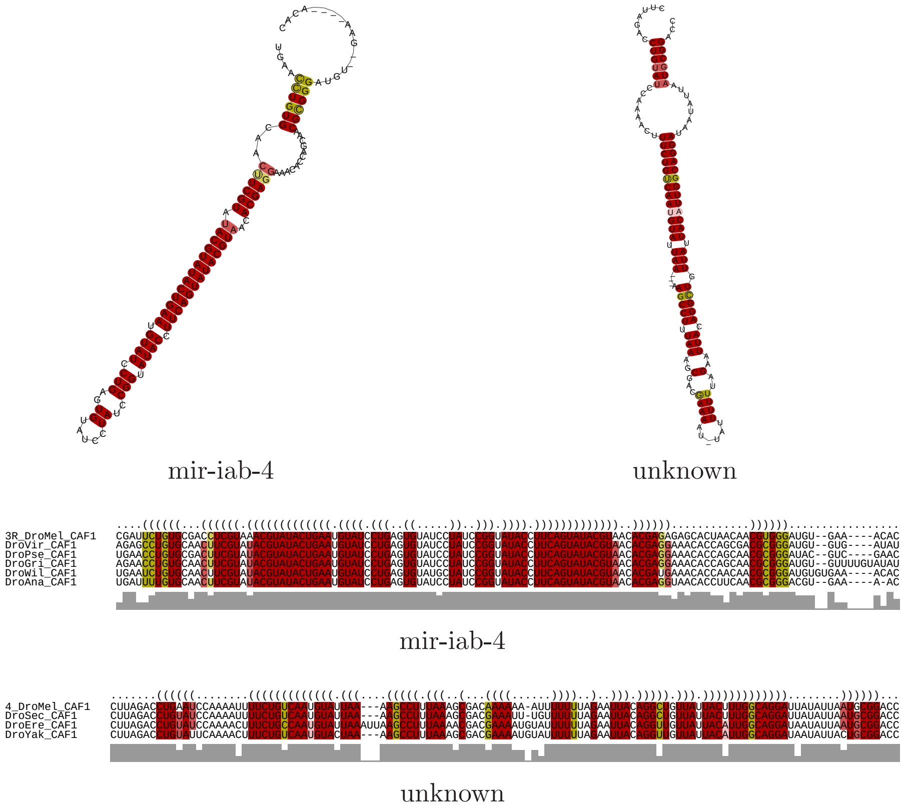

Figure 3: Exemplary consensus secondary structures of two `RNAz` predictions.
The left structure shows the high-scoring ($p > 0.99$) locus 4842 of chromosome 3R ranging from 12 681 981 to 12 682 095. It coveres the well known miRNA mir-iab-4, which is already available at the `Noncode`, the `miRBase` or `FlyBase`. In turn, we present a putative novel miRNA structure found at locus 132 of chromosome 4 (599 336 - 599 452) at the right. The `RNAz` p-value is 0.99 and even `RNAmicro` scored this locus with 0.99.

DroMel_Locus	Human	Urochordates	Nematodes	Annotation
2L_locus2914	10.5	-	-	U6atac_snRNA
2L_locus6302	6.8	5.1	-	dme-mir-124
2L_locus7421	6.8	-	-	dme-mir-133
3L_locus7298	7.0	-	-	dme-mir-9a
2R_locus4945	5.7	5.7	-	dme-mir-7
3L_locus6533	5.0	-	-	dme-mir-219
3L_locus5305	-	-	5.0	-
2R_locus5718	5.2	-	-	-
3L_locus6232	5.8	-	-	TC-rich low complexity
2R_locus4961	-	5.1	-	-
3L_locus8562	-	-	5.9	*C. briggsae*
X_locus6764	7.5	-	-	-
3L_locus7318	+	-	-	CG8786-RB; RNAmicro_p:0.972748
2L_locus3569	11.7	7.5	-	knownCDS=RpS27A-cds
2R_locus213	-	5.7	-	knownCDS=Act42A-cds
2R_locus5064	6.8	34.7	-	actin CDS [also in *C. intestinalis*
2R_locus5066	6.3	-	-	actin CDS [also in *C. intestinalis*]
2R_locus6548	6.1	-	-	RPL-19 gene/pseudogene
2R_locus666	7.9	-	-	knownCDS=Gapdh1-cds
X_locus2261	5.1	-	-	knownCDS=CG32744

Table 1: Patterns of conserved RNAs
Patterns of conserved RNAs revealed by `BLAST` searches of the drosophilid `RNAz` hits against the results of prior `RNAz` surveys regarding mammals [1], urochordates [2], and nematodes [3]. Denoted numerical table entries are $-log(eValue)$. The U6atac snRNA and 5 microRNAs are recovered.

		chromosomes					
	all	2L	2R	3L	3R	4	X
`RNAz` loci ($p > 0.5$, normal screen)	42 482	7 824	6 646	8 765	10 351	196	8 700
`RNAz` loci ($p > 0.5$, control screen)	24 018	4 266	3 802	5 038	5 791	134	4 987
(a) FDR ($p > 0.5$, [%])	56.53	54.52	57.20	57.47	55.94	68.36	57.32
loci ($p > 0.5$, [kb], normal screen)	5 079	927	783	1 060	1 229	25	1 055
loci ($p > 0.5$, [kb], control screen)	2 680	470	420	571	644	16	559
(b) FDR ($p > 0.5$, [%])	52.76	50.70	53.63	53.86	52.40	64	52.98
`RNAz` loci ($p > 0.9$, normal screen)	16 377	2 940	2 473	3 413	3 862	80	3 609
`RNAz` loci ($p > 0.9$, control screen)	7 427	1 281	1 115	1 631	1 784	35	1 581
(a) FDR ($p > 0.9$, [%])	45.35	43.57	45.08	47.78	46.19	43.75	43.80
loci ($p > 0.9$, [kb], normal screen)	2 167	385	321	461	511	11	478
loci ($p > 0.9$, [kb], control screen)	871	147	129	196	210	4	185
(b) FDR ($p > 0.9$, [%])	40.19	38.18	40.18	42.51	41.09	36.36	38.70

Table 2: Comparison of `RNAz` predicted ncRNAs using normal and randomized alignments.
We counted the number of predicted loci and their overall length at two probability thresholds ($p > 0.5$, $p > 0.9$) for normal and also randomized alignments. Obtained relative frequencies may be interpreted as false discovery rates (FDR). As expected, the FDR decreases with a higher `RNAz` p-value.

		chromosomes					
	all	2L	2R	3L	3R	4	X
`RNAz` loci ($p > 0.5$)	42 482	7 824	6 646	8 765	10 351	196	8 700
`RNAz` windows	68 562	12 540	10 587	14 342	16 476	318	14 299
`RNAz` loci ($p > 0.9$)	16 377	2 940	2 473	3 413	3 862	80	3 609
`RNAz` windows	23 926	4 353	3 621	5 028	5 596	117	5 211
loci ($p > 0.5$, [kb])	5 036	919	776	1 052	1 218	25	1 046
annotated	8 773	1 609	1 610	1 718	2 133	70	1 633
[%]	20.7	20.6	24.2	19.6	20.6	35.7	18.8
unannotated	33 709	6 215	5 036	7 047	8 218	126	7 067
[%]	79.3	79.4	75.8	80.4	79.4	64.3	81.2
`tRNAscan`	159	21	57	30	46	0	5
`RNAmicro`	607	114	113	120	122	4	134
`SnoReport`	59	8	10	15	13	0	13
`Rfam`	222	47	67	36	62	0	10
`Noncode`	44	25	7	1	8	0	3
`ncRNAdb`	89	35	18	3	22	0	11
`FlyBase`	316	75	87	46	89	0	19
`miRBase`	79	20	22	13	18	0	6
tRNA	171/250/297 (69 %)	23/36/41	60/83/100	31/43/49	50/68/80	-	7/20/27
rRNA_5S	0/0/99 (100 %)	-	0/0/99	-	-	-	-
RNase_P_RNA	1/1/1 (100 %)	-	-	-	1/1/1	-	-
SRP_RNA	0/0/2 (100 %)	-	-	-	0/0/2	-	-
U1_snRNA	5/5/5 (100 %)	1/1/1	-	-	4/4/4	-	-
U2_snRNA	5/5/5 (100 %)	5/5/5	-	-	-	-	-
U4_snRNA	2/3/3 (67 %)	2/3/3	-	-	-	-	-
U5_snRNA	6/6/6 (100 %)	5/5/5	-	1/1/1	-	-	-
U6_snRNA	0/3/3 (0 %)	-	-	-	0/3/3	-	-
snoRNA	96/202/250 (48 %)	8/27/39	20/74/84	11/36/48	16/37/44	-	14/28/35
miRNA	75/78/85 (96 %)	19/20/21	22/22/26	14/15/16	15/15/15	-	5/6/7
known CDS ($p > 0.5$)	8 021	1 449	1 452	1 588	1 960	66	1 506
known CDS ($p > 0.9$)	2 208	374	339	451	552	19	473
in `RNAz` input	58 076	10 477	11 853	10 897	14 711	935	9 203
generally known	69 245	12 444	14 149	12 835	17 391	1 097	10 780
at introns ($p > 0.5$)	13 712	2 327	2 264	2 921	3 332	77	2 791
[%]	32.3	29.7	34.1	33.3	32.2	39.3	32.1
at introns ($p > 0.9$)	5 602	908	923	1 224	1 292	35	1 220
[%]	34.2	30.9	37.3	35.9	33.5	43.8	33.8

Table 3: Summary of `RNAz` predicted ncRNAs.
At least one or more overlapping `RNAz` windows form exactly one `RNAz` locus. Those loci are the actual set of predicted ncRNA candidates ($p > 0.5$). We denote the number of `tRNAscan`, `RNAmicro` and `SnoReport` hits within our predictions. Furthermore, we state the number of annotatable predictions obtained by `BLAST` searches against several ncRNA providing databases. Moreover, we count the number of recovered already annotated *D. melanogaster* ncRNAs. Thereby, we state three '/'-separated numbers representing (1) the retrieved number of known ncRNAs, (2) the actual amount of those known elements in the `RNAz` input alignments and (3) their overall sum in general. Additionaly, we list the number of `RNAz` hits associated with CDS elements and introns. Congruously, only elements included in the alignment input set are retrievable. This percentage, additionally, is given in the column 'overall'. For example, the `RNAz` input comprises 250 tRNAs and the prediction yielded 171 ncRNA loci what actually is 69 % of all 297 well-known tRNAs. Obviously, not every chromosome contains each of the given elements ('-'). An overlap of at least 70 % is required during the annotation of `RNAz` hits with known elements. The 5S rRNAs are located at a cluster ranging from 1 524 441 to 15 281 141 at chromosome 2R. Unfortunately, none of the latter are within the selected `RNAz` input alignments, thus, we are not able to find them. A mean pairwise identity of 100 % for the aligned U6 snRNAs elucidates why `RNAz` classification fails for this ncRNA. A comprehensible amount of predictions is located at introns.

	Timepoint												
	all	1	2	3	4	5	6	7	8	9	10	11	12
`RNAz` loci (1)	4236	2544	2491	1622	2779	3068	3334	2250	2359	2657	1710	1844	2107
`RNAz` loci (2)	2655	1719	1621	1083	1759	1956	2086	1492	1565	1691	1138	1217	1370
Enrichment	1.59	1.48	1.53	1.50	1.59	1.56	1.60	1.51	1.51	1.57	1.50	1.51	1.54
`RNAz` loci (3)	1713	836	850	574	993	1120	1284	831	919	1035	655	697	826
`RNAz` loci (4)	891	510	497	328	565	642	680	468	472	532	342	380	426
Enrichment	1.92	1.63	1.71	1.75	1.76	1.74	1.77	1.94	1.95	1.91	1.83	1.94	1.92

Table 4: Number of predicted ncRNAs which overlap with Transfrags from [4].
(1)=($p > 0.5$, normal screen); (2)=($p > 0.5$, control screen); (3)=($p > 0.9$, normal screen); (4)=($p > 0.9$, control screen)

	Number of timepoints											
	all	1	2	3	4	5	6	7	8	9	10	11
`RNAz` loci (1)	478	912	668	546	408	427	401	350	300	297	241	275
`RNAz` loci (2)	359	523	377	299	265	243	207	225	179	191	176	200
Enrichment	0.75	0.99	1.00	1.03	0.87	0.99	1.09	0.88	0.95	0.88	0.77	0.78
`RNAz` loci (3)	153	528	343	229	171	173	145	133	105	94	90	85
`RNAz` loci (4)	101	251	151	105	115	84	71	74	58	51	42	53
Enrichment	0.69	0.95	1.03	0.99	0.67	0.93	0.93	0.81	0.82	0.83	0.97	0.73

Table 5: Number of predicted ncRNAs which overlap with Transfrags from [4] in one, several or all timepoints.
Enrichments are calculated based on the fraction of overlapping hits and the respective number of total hits. (1)=($p > 0.5$, normal screen); (2)=($p > 0.5$, control screen); (3)=($p > 0.9$, normal screen); (4)=($p > 0.9$, control screen)

	$p > 0.5$	$p > 0.9$
Genes and Gene prediction Tracks		
FlyBase	8 524	2 558
RefSeq	8 625	2 598
N-SCAN	6 691	1 806
Genscan	7 598	2 175
Human Proteins	1 110	303
mRNA and EST Tracks		
mRNA	7 431	2 282
EST	7 865	2 623
RepeatMasker	5	3

Table 6: Intersection ($> 80\,\%$) of `RNAz` predictions and `UCSCS Table Browser` tracks.

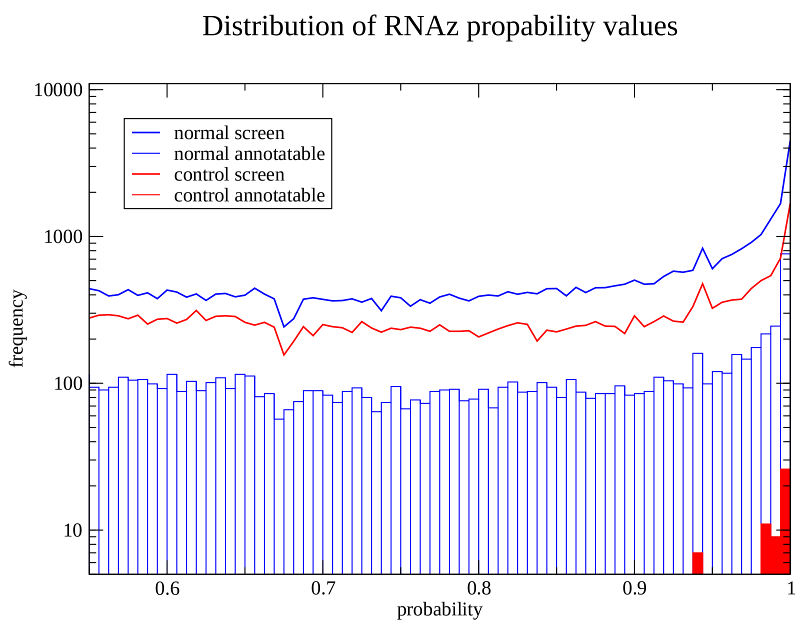

Figure 4: Comparison of obtained p-values.
The figure illustrates the distribution of resulting `RNAz` probability values (p-values) of the complete `RNAz` candidate set (straight lines, top) compared to the restricted set of annotatable candidates (bar-representation, bottom). This is done for both, the normal (blue) and the control screen (red). The y-axis is scaled logarithmically. Promisingly, the number of high-scoring predictions of the normal screen prevails and, fortunately, most of those ncRNA candidates yielding high p-values are annotatable. Although a substantial fraction of hits is obtained using randomized alignments, the majority of the latter lacks an annotation. However, this is indication for a trustable `RNAz` scoring scheme but demonstrates the necessity of annotating the predictions.

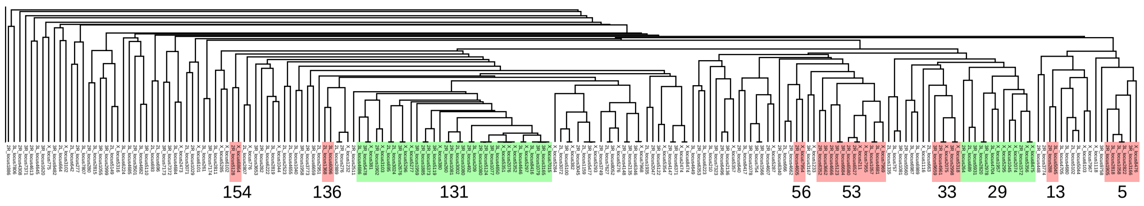

Figure 5: Complete WPGMA cluster tree of RNA candidates overlapping TRF and BRF binding regions.
The clustering tree was created by agglomerative clustering where the distances correspond directly to the `LocARNA` alignment score [5]. To avoid that large scores influence the distance transformation we define distances by $d(i,j) = max(0, q - score(i,j))$, where q is here the 99% quantil of all pairwise scores. Most prominent clusters are highlighted red. Detailed information about those clusters is given in Table 7. Clusters 131 and 29 (highlighted green) are described in main text.

Cluster	N	Score	MPI	SCI	MFE	Length	Consensus
56	2	4664	21.74	1.40	-26.80	92	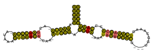
13	2	4746	25.00	1.32	-28.35	84	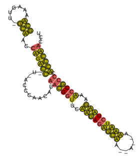
154	2	4055	32.63	1.29	-22.00	95	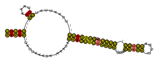
136	2	5041	22.78	1.19	-23.25	79	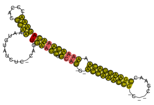
5	6	3004	26.10	0.71	-13.90	89	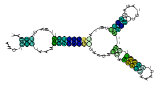
33	5	2905	29.28	0.74	-16.19	94	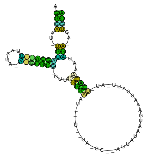
53	12	1743	22.00	0.76	-7.42	46	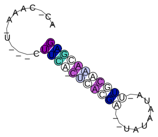

Table 7: Most prominent structural clusters of novel RNA candidates that overlap TRF or BRF binding regions.

In the upper part of the table the most structural conserved clusters are shown. The lower part depicts cluster with larger number of sequences but still high structural conservation.

N...number of sequences in cluster. Score...`MLocARNA` score of multiple alignment of cluster sequences. MPI...mean pairwise identity of multiple alignment. SCI...structural conservation index. MFE...minimum free energy of consensus secondary structure of multiple alignment. Length...alignment length. Consensus...consensus secondary structure of alignment.

References

[1] S. Washietl, I. L. Hofacker, and P. F. Stadler. Fast and reliable prediction of noncoding RNAs. *Proc. Natl. Acad. Sci. USA*, 102:2454–2459, 2005.

[2] K. Missal, D. Rose, and P. F. Stadler. Non-coding RNAs in Ciona intestinalis. *Bioinformatics*, 21 Suppl 2:ii77–ii78, Sep 2005.

[3] K. Missal, X. Zhu, D. Rose, W. Deng, G. Skogerbo, R. Chen, and P. F. Stadler. Prediction of structured non-coding RNAs in the genomes of the nematodes Caenorhabditis elegans and Caenorhabditis briggsae. *J Exp Zoolog B Mol Dev Evol*, Jan 2006.

[4] J. R. Manak, S. Dike, V. Sementchenko, P. Kapranov, F. Biemar, J. Long, J. Cheng, I. Bell, S. Ghosh, A. Piccolboni, and T. R. Gingeras. Biological function of unannotated transcription during the early development of Drosophila melanogaster. *Nat Genet*, 38(10):1151–1158, Oct 2006.

[5] S. Will, K. Reiche, I. L. Hofacker, P. F. Stadler, and R. Backofen. Inferring Non-Coding RNA families and classes by means of structure-based clustering. 2007. submitted.
